# NIR-Responsive Gold-Decorated Phase-Change Nanodroplets for Photothermal-Triggered Pulsatile Doxorubicin Release and Enhanced Combined Photothermal-Chemotherapy in Triple-Negative Breast Cancer

**DOI:** 10.3390/pharmaceutics18070816

**Published:** 2026-06-30

**Authors:** Luyao Ma, Fulai Chen, Qinghao Xu, Jianwei Yu, Yang Liu, Lei Duan

**Affiliations:** School of Biomedical Engineering and Informatics, Nanjing Medical University, Nanjing 211166, China; luyao@stu.njmu.edu.cn (L.M.);

**Keywords:** triple-negative breast cancer, phase-change nanodroplet, photothermal therapy, controlled drug release

## Abstract

**Background**: Triple-negative breast cancer (TNBC), devoid of actionable targets for endocrine or HER2-directed therapy, is highly aggressive with elevated risks of recurrence and metastasis; surgical resection remains the mainstay of treatment, and postoperative chemotherapy serves as a key adjuvant modality for controlling residual disease. Doxorubicin (DOX), although widely used, shows limited tumor selectivity, considerable systemic toxicity, and poor control over drug release at the tumor site. To address these issues, we developed near-infrared (NIR)-responsive gold-decorated phase-change nanodroplets (AuNPs-DOX-NDs) that combine photothermal conversion, liquid-to-gas phase transition, and controlled DOX release in a single platform. **Methods**: The nanodroplets consisted of a perfluorohexane (PFH) core, a DOX-loaded lipid shell, and polyethyleneimine-modified gold nanoparticles (PEI-AuNPs) conjugated to the surface as the NIR photothermal component. Physicochemical characterization was performed to evaluate morphology, colloidal dispersity, and storage stability. Under 808 nm laser irradiation, the photothermal behavior, PFH vaporization, and DOX release properties of AuNPs-DOX-NDs were investigated. In vitro studies using 4T1 TNBC cells were conducted to assess intracellular DOX accumulation, cell proliferation, migration, and apoptosis. **Results**: Physicochemical characterization showed that the nanodroplets had a uniform nanoscale morphology, good colloidal dispersity, and acceptable storage stability. Under 808 nm laser irradiation, AuNPs-DOX-NDs exhibited concentration-dependent photothermal heating, which induced PFH vaporization and accelerated DOX release, indicating a clear stimulus-responsive release behavior. In vitro studies using 4T1 TNBC cells showed enhanced intracellular DOX accumulation after treatment with AuNPs-DOX-NDs. Upon laser irradiation, the nanodroplets further inhibited cell proliferation and migration and promoted apoptosis, suggesting an enhanced combined photothermal–chemotherapeutic effect in 4T1 TNBC cells. **Conclusions**: These results indicate that AuNPs-DOX-NDs may serve as a useful NIR-responsive platform for externally controlled drug release and enhanced combined photothermal-chemotherapy, and deserve further evaluation in vivo.

## 1. Introduction

Triple-negative breast cancer (TNBC) is an aggressive subtype of breast cancer characterized by the lack of estrogen receptor, progesterone receptor, and human epidermal growth factor receptor 2 expression [1,2]. Because of the absence of effective therapeutic targets, endocrine therapy and HER2-targeted therapy are not applicable to TNBC, and systemic chemotherapy remains a standard treatment strategy [3,4,5]. However, improving local treatment efficacy, increasing drug accumulation in tumors, and reducing off-target toxicity remain major challenges in TNBC therapy [4,6].

Doxorubicin (DOX) is a commonly used anthracycline drug in breast cancer treatment. Its antitumor activity mainly arises from DNA intercalation, topoisomerase II inhibition, and oxidative stress induction [7,8]. However, free DOX shows poor selectivity in vivo and often causes serious adverse effects, including cardiotoxicity and myelosuppression. In addition, the release of free DOX at tumor sites cannot be precisely controlled, which limits its therapeutic benefit [7,8,9]. These drawbacks suggest that DOX alone is insufficient for the precise treatment of TNBC and highlight the need for drug delivery systems that can improve tumor accumulation and allow controlled release.

Phase-change nanodroplets (NDs) are usually composed of a liquid perfluorocarbon core surrounded by a lipid or polymer shell. Owing to their nanoscale size, they can accumulate in tumors through the enhanced permeability and retention effect [10]. After external stimulation, the liquid core can undergo a liquid-to-gas transition, accompanied by volume expansion and shell disruption, thereby promoting rapid drug release [11]. Compared with conventional diffusion-based nanocarriers, phase-change nanodroplets offer more responsive and amplified release behavior [12,13,14]. However, a simple DOX-NDs system still has two main limitations. First, it does not generate heat by itself, so phase transition usually depends on external heating or high-intensity focused ultrasound, which may be difficult to control precisely in vivo. Second, chemotherapy alone provides only one treatment mode, which may not be sufficient for effective TNBC treatment.

To address these limitations, the introduction of a near-infrared (NIR) photothermal component into phase-change nanodroplets is a promising approach. Gold nanoparticles (AuNPs) show localized surface plasmon resonance and can efficiently convert NIR light into heat [15,16]. When incorporated into drug-loaded phase-change nanodroplets, AuNPs can provide several advantages. First, photothermal heating induced by AuNPs can directly damage tumor cells. Second, the generated heat can trigger PFH vaporization and promote pulsatile DOX release, allowing externally controlled drug delivery. Third, the combination of photothermal therapy and chemotherapy may improve the overall antitumor effect, especially in aggressive tumors such as TNBC [17,18,19,20]. Therefore, AuNP incorporation may compensate for the lack of active triggering in the DOX-ND system and also expand it from a single therapeutic mode to a combined treatment strategy.

Although AuNP-based phase-change nanocarriers have been reported previously, directly comparable systems that simultaneously integrate gold nanostructures, perfluorocarbon phase-change droplets, and chemotherapeutic drug delivery remain relatively limited, especially when compared with the much more established field of ultrasound-responsive phase-change droplets. In many previous studies, gold nanostructures have mainly been used as co-encapsulated photothermal/photoacoustic components or as shell-forming materials to facilitate laser-induced vaporization. By contrast, in the present study, PEI-AuNPs were conjugated onto the surface of PFH-core, DOX-loaded lipid nanodroplets as an interface-localized photothermal functional layer. This design enables AuNPs to act not merely as photothermal agents, but more importantly as external energy-conversion mediators that transform 808 nm NIR light into localized heat at the droplet interface and thereby regulate the phase-transition behavior of the PFH core. Because phase-change droplets are intrinsically regulatable carriers whose internal structure can be altered by external energy input, such surface functionalization introduces a new light-responsive control mode in addition to the more commonly used ultrasound-triggered activation. Therefore, the distinctive feature of the present system is not simply the incorporation of AuNPs, but the use of surface AuNP-mediated photothermal conversion to actively control PFH phase transition and achieve externally triggered, pulsatile DOX release.

**Figure 1:**
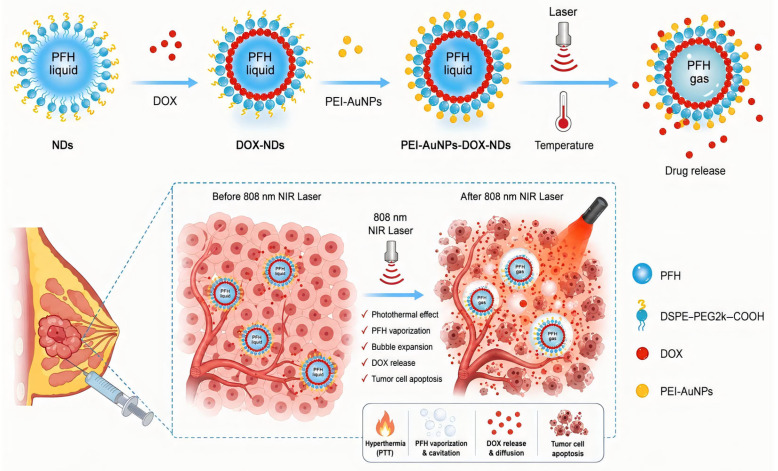
Construction and regulatory mechanism of the gold-decorated phase-change nanodroplet drug delivery system.

Based on this rationale, we constructed gold-decorated phase-change nanodroplets (AuNPs-DOX-NDs) consisting of a PFH core, a DOX-loaded lipid shell, and surface-conjugated PEI-AuNPs introduced through EDC/NHS coupling as an interface-localized photothermal layer for NIR-triggered regulation of PFH phase transition. In vitro results showed that under 808 nm laser irradiation, AuNPs-DOX-NDs achieved photothermal conversion, triggered PFH vaporization, promoted pulsatile DOX release, and produced an enhanced combined photothermal–chemotherapeutic effect in 4T1 TNBC cells after laser activation (Figure 1).

## 2. Materials and Methods

### 2.1. Materials

1,2-Dipalmitoyl-sn-glycero-3-phosphocholine (DPPC) and 1,2-distearoyl-sn-glycero-3-phosphoethanolamine-N-carboxy-polyethylene glycol 2000 (DSPE-PEG2000-COOH) were purchased from Aladdin Biochemical Technology Co., Ltd. (Shanghai, China). Perfluorohexane (PFH) and doxorubicin (DOX) were obtained from Shanghai Macklin Biochemical Technology Co., Ltd. (Shanghai, China). Polyethyleneimine-modified gold nanoparticles (PEI-AuNPs, 15 nm) and MES buffer were purchased from Nanjing Dongna Biotechnology Co., Ltd. (Nanjing, China). 1-Ethyl-3-(3-dimethylaminopropyl) carbodiimide hydrochloride (EDC·HCl) and N-hydroxysuccinimide (NHS) were purchased from Sinopharm Chemical Reagent Co., Ltd. (Shanghai, China). Methanol and all other reagents were of analytical grade and used without further purification.

### 2.2. Preparation of Phase-Change Nanodroplets

#### 2.2.1. Preparation of Blank and Drug-Loaded Nanodroplets (NDs and DOX-NDs)

Blank phase-change nanodroplets (NDs) were prepared using a reciprocating differential-pressure method. Briefly, 4.0 mL of buffer solution (0.9% saline containing 10% glycerol and 4% ethanol, *v*/*v*) was added to a sealed vial containing DPPC (0.8 mg) and DSPE-PEG2000-COOH (9.16 mg). The mixture was sonicated in a water bath until the lipids were completely dissolved. After replacement of the headspace air with PFH gas, 3.0 mL of PFH was injected into the vial. The vial was then subjected to 100 cycles of manual reciprocating compression, and the resulting emulsion was immediately cooled in an ice bath. The obtained NDs were purified by centrifugation (8000 rpm, 10 min, 4 °C) and washed three times with ultrapure water. DOX-loaded nanodroplets (DOX-NDs) were prepared in the same manner, except that DOX was added to the initial buffer solution at a final concentration of 0.20 mg/mL.

#### 2.2.2. Preparation of Gold-Loaded Nanodroplets (AuNPs-DOX-NDs)

To prepare AuNPs-DOX-NDs, the surface carboxyl groups of DOX-NDs were first activated by EDC/NHS coupling. Purified DOX-NDs were resuspended in MES buffer (0.1 M, pH 5.5). EDC (120 mg) and NHS (180 mg) were added, and the mixture was shaken at 25 °C for 15 min. PEI-AuNPs solution (1.0 mL, 1 mg/mL) was then added, and the reaction was allowed to proceed at 25 °C in the dark for 24 h with gentle shaking. The resulting AuNPs-DOX-NDs were purified by centrifugation (8000 rpm, 10 min, 4 °C) and washed three times to remove unreacted reagents and free AuNPs.

### 2.3. Characterization of Nanodroplets

#### 2.3.1. Particle Size, Zeta Potential, and Stability

The hydrodynamic diameter, polydispersity index (PDI), and zeta potential of NDs, DOX-NDs, and AuNPs-DOX-NDs were measured at 25 °C using a dynamic light scattering (DLS) analyzer (Litesize 500, Anton Paar, Graz, Austria). To evaluate stability, the particle size and PDI of AuNPs-DOX-NDs stored at 4 °C were monitored over 7 days. All measurements were performed in triplicate.

#### 2.3.2. Morphological Observation

The morphology of the nanodroplets was observed using transmission electron microscopy (TEM) (JEM-1400Flash, JEOL, Tokyo, Japan). Diluted samples (5 μL) were deposited onto carbon-coated copper grids, air-dried, and observed at an accelerating voltage of 200 kV.

#### 2.3.3. Determination of Drug Loading Content and Encapsulation Efficiency

The absorbance of DOX was measured at 480 nm using a UV–Vis spectrophotometer (UH5300, Hitachi, Tokyo, Japan). A standard calibration curve was established using DOX solutions of known concentrations. To determine drug loading, a known volume of AuNPs-DOX-NDs was disrupted with methanol, followed by centrifugation. The absorbance of the resulting supernatant was measured. Encapsulation efficiency (EE%) and drug loading capacity (LC%) were calculated according to the following equations:

(1) $$EE\%=\frac{\mathrm{weight}\;\mathrm{of}\;\mathrm{DOX}\;\mathrm{in}\;\mathrm{NDs}}{\mathrm{weight}\;\mathrm{of}\;\mathrm{initially}\;\mathrm{added}\;\mathrm{DOX}}\times 100\%$$

(2) $$LC\%=\frac{\mathrm{weight}\;\mathrm{of}\;\mathrm{DOX}\;\mathrm{in}\;\mathrm{NDs}}{\mathrm{weight}\;\mathrm{of}\;\mathrm{NDs}}\times 100\%$$

#### 2.3.4. Quantification of Gold Loading

The Au content in AuNPs-DOX-NDs was determined by inductively coupled plasma optical emission spectrometry (ICP-OES) (iCAP PRO, Thermo Fisher, Waltham, MA, USA). A known volume of the nanodroplet suspension was digested with freshly prepared aqua regia at 80 °C. The digest was then diluted appropriately, and Au was quantified at 242.795 nm.

### 2.4. Evaluation of the Photothermal Performance of AuNPs-DOX-NDs

The photothermal properties of AuNPs-DOX-NDs were evaluated by irradiating 200 μL of sample suspension (1 × 10^8^ particles/mL) in a 96-well plate with an 808 nm NIR laser (MDL-D-808/0-2W, CNI, Changchun, China) at different power densities (0.5, 1.0, 1.5, and 2.0 W/cm^2^) for 15 min. The laser spot diameter was 5 mm, and the distance from the fiber tip to the sample was 5 cm. The sample temperature was monitored in real time using an infrared thermal imaging camera (FLIR ONE Pro, Wilsonville, OR, USA). Free PEI-AuNPs at the same Au concentration were used as a control. Photothermal stability was further assessed over three laser on/off cycles.

### 2.5. Temperature- and Laser-Regulated Phase Transition and Drug Release

#### 2.5.1. Temperature-Responsive Behavior

The phase-transition behavior of NDs and AuNPs-DOX-NDs was examined by placing the samples on a temperature-controlled stage at room temperature, 37 °C, 56 °C, 65 °C, and 70 °C. Changes in particle size distribution were analyzed by DLS, and morphological changes were observed under an optical microscope (AMEX1000, Thermo Fisher, USA).

#### 2.5.2. Laser-Responsive Behavior

To evaluate the response to laser irradiation, NDs and AuNPs-DOX-NDs were exposed to an 808 nm laser (2.0 W/cm^2^) for different durations (1, 2, 4, and 6 min). Changes in particle size and morphology were then analyzed as described in Section 2.5.1.

#### 2.5.3. In Vitro Drug Release Study

For temperature-dependent release, AuNPs-DOX-NDs suspensions were incubated at 25 °C, 37 °C, 56 °C, 65 °C, or 70 °C. At predetermined time points within 5 h, samples were collected and centrifuged, and the released DOX in the supernatant was quantified by UV–Vis spectroscopy.

For laser-triggered release, AuNPs-DOX-NDs were divided into three groups: a control group, laser group A, and laser group B. The control group was incubated at 37 °C without laser irradiation and sampled at the same time points as the laser-treated groups to determine the baseline release profile. Laser group A was sampled immediately before each laser pulse at t = 0, 1, 2, 3, 4, 5, …, 30 h, whereas laser group B was sampled immediately after each laser pulse, namely at 15 min, 1 h 15 min, 2 h 15 min, 3 h 15 min, 4 h 15 min, 5 h 15 min, …, 30 h 15 min. Each group at each time point included five independent parallel samples (*n* = 5).

Laser irradiation was performed in an intermittent pulse mode. At each full-hour time point from 0 to 30 h, the laser-treated samples were irradiated using an 808 nm NIR laser at a power density of 2.0 W/cm^2^ for 15 min. The irradiation distance was fixed at 5 cm, and the laser spot diameter was 5 mm. The control samples were placed under the same environmental conditions but were not exposed to laser irradiation. At each sampling point, 0.2 mL of sample was collected and centrifuged at 5000 rpm for 3 min. The supernatant was collected as the released/free DOX fraction, and its absorbance was measured at 480 nm using a UV–Vis spectrophotometer. The DOX concentration was calculated according to a pre-established standard calibration curve. The released DOX mass was calculated by multiplying the measured DOX concentration by the remaining release-medium volume after sampling, and the cumulative drug release efficiency was then calculated to analyze the release behavior of AuNPs-DOX-NDs.

### 2.6. In Vitro Biological Evaluation

#### 2.6.1. Cell Culture

Murine breast cancer 4T1 cells were obtained from the American Type Culture Collection (ATCC). The cells were cultured in DMEM supplemented with 10% fetal bovine serum (FBS) and 1% penicillin–streptomycin at 37 °C in a humidified incubator containing 5% CO_2_.

#### 2.6.2. In Vitro Biocompatibility, Laser Safety, and Cellular Uptake

The cytotoxicity of the blank carrier (AuNPs-NDs) was evaluated by the CCK-8 assay. Briefly, 4T1 cells were seeded in 96-well plates and incubated with different concentrations of AuNPs-NDs (0–100 μg/mL) for 24 h. Cell viability was then measured, and the maximum safe carrier concentration was determined for subsequent experiments. The effect of laser irradiation alone on cell viability was also assessed using the CCK-8 assay. In the absence of any material treatment, cells were exposed to an 808 nm laser (1 W/cm^2^) for 0, 2, 4, 6, 8, or 10 min and then further cultured for 24 h before viability measurement. For the cellular uptake study, 4T1 cells were incubated with each formulation for 24 h. Intracellular DOX fluorescence was observed by confocal laser scanning microscopy, and the uptake level was further quantified by flow cytometry.

#### 2.6.3. In Vitro Antitumor Efficacy

Four treatment groups were included AuNPs-NDs, free DOX, DOX-NDs, and AuNPs-DOX-NDs. AuNPs-NDs, DOX-NDs, and AuNPs-DOX-NDs were applied at the maximum safe carrier concentration determined in Section 2.6.2, while free DOX was added at the equivalent DOX concentration corresponding to that carrier dose.

The in vitro antitumor effects were evaluated using the CCK-8 assay, morphological observation, colony formation assay, apoptosis analysis, and wound healing assay. Each treatment group was further divided into laser and non-laser subgroups. Cells in the non-laser subgroup were incubated for 24 h after treatment. Cells in the laser subgroup were irradiated with an 808 nm laser (1 W/cm^2^, 4 min) at 2 h after treatment and then incubated for the remaining time within the 24 h period. The irradiation condition of 1.0 W/cm^2^ for 4 min was selected based on preliminary laser safety and photothermal optimization experiments in the cell culture system. Under this condition, laser irradiation alone did not induce obvious cytotoxicity, while AuNPs-DOX-NDs produced a moderate temperature increase suitable for photothermal activation. All subsequent assays were performed after treatment. For the colony formation assay, cells were seeded in 6-well plates at a density of 1000 cells per well, treated as described above, and cultured for 10 days. The colonies were then fixed, stained with crystal violet, and counted. For apoptosis analysis, cells were stained with Annexin V-FITC/PI and analyzed by flow cytometry. For the wound healing assay, cells were grown to confluence and scratched with a pipette tip. The corresponding treatments were then applied. Images were recorded at 0 and 24 h after treatment, and the wound closure rate was calculated.

### 2.7. Statistical Analysis

All data are presented as mean ± standard deviation (SD). Statistical comparisons among multiple groups were performed using one-way analysis of variance (ANOVA) followed by Tukey’s post hoc multiple-comparison test. A *p* value < 0.05 was considered statistically significant. Statistical significance was indicated as * *p* < 0.05, ** *p* < 0.01, and *** *p* < 0.001.

## 3. Results

### 3.1. Preparation and Characterization of Gold-Loaded Phase-Change Nanodroplets

**Figure 2:**
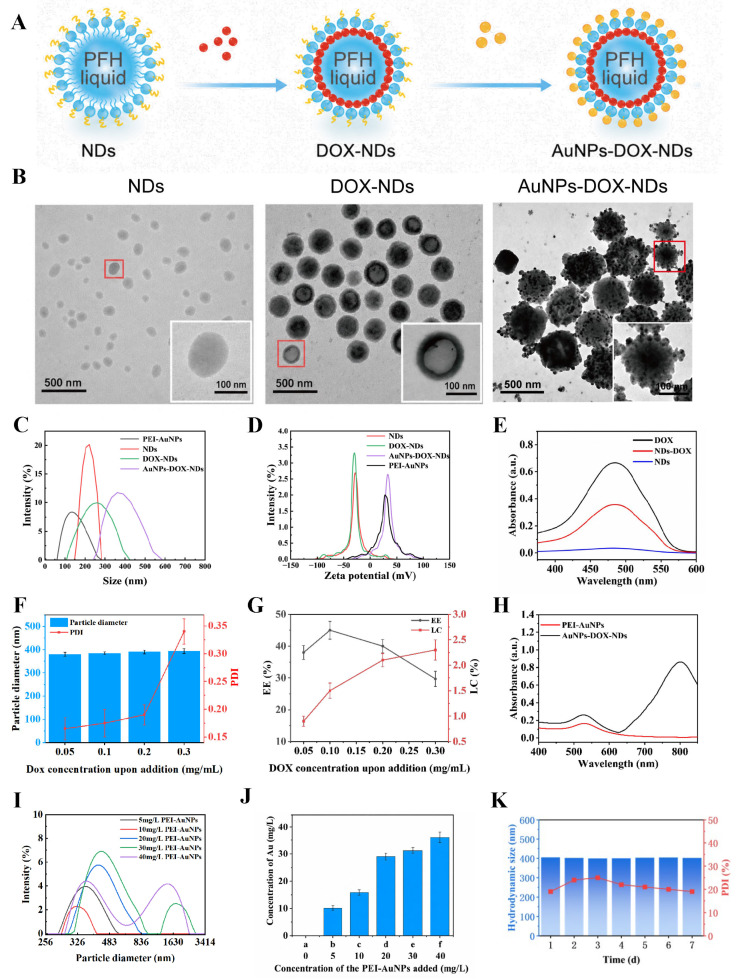
Preparation and characterization of AuNPs-DOX-NDs. (**A**) Schematic illustration of the construction process. (**B**) Representative TEM images of NDs, DOX-NDs, and AuNPs-DOX-NDs, corresponding to higher-magnification images are shown as insets (main images, scale bar = 500 nm; insets, scale bar = 100 nm). (**C**) Particle size distribution curves of PEI-AuNPs, NDs, DOX-NDs, and AuNPs-DOX-NDs. (**D**) Zeta potential distribution of NDs, DOX-NDs, AuNPs-DOX-NDs, and PEI-AuNPs. (**E**) UV–Vis absorption spectra of DOX and DOX-NDs, with 480 nm as the characteristic DOX absorption peak. (**F**) Particle size and PDI changes in DOX-loaded nanodroplets at different initial DOX concentrations. (**G**) Encapsulation efficiency and drug loading content at different initial DOX concentrations. (**H**) UV–Vis absorption spectra comparison of AuNPs-DOX-NDs and PEI-AuNPs. (**I**) Particle size distribution curves of AuNPs-DOX-NDs at different PEI-AuNP addition concentrations. (**J**) ICP-OES quantification of Au content in AuNPs-DOX-NDs at different PEI-AuNP concentrations. (**K**) Hydrodynamic particle size and PDI changes in AuNPs-DOX-NDs over 7 days.

Blank nanodroplets (NDs) with a PFH core were first prepared using a reciprocating differential-pressure method. DOX was then encapsulated to obtain DOX-NDs, followed by conjugation of PEI-AuNPs onto the droplet surface through EDC/NHS coupling to generate AuNPs-DOX-NDs (Figure 2A).

TEM images showed that NDs, DOX-NDs, and AuNPs-DOX-NDs all had relatively uniform spherical vesicle-like structures (Figure 2B). Compared with blank NDs, DOX-NDs exhibited a clearer ring-like shell boundary, which may be related to the lipid-shell structure and the increased electron density at the interface after DOX loading. After PEI-AuNPs conjugation, AuNPs-DOX-NDs still maintained an intact vesicular morphology, but discrete electron-dense nanoparticles appeared on the shell surface instead of the continuous ring-like boundary seen in DOX-NDs, suggesting successful surface attachment of PEI-AuNPs.

According to DLS analysis, the average particle sizes of blank NDs and PEI-AuNPs were 211.57 ± 3.84 nm and 101.57 ± 3.61 nm, respectively (Figure 2C). After DOX loading, the particle size increased to 251.77 ± 13.11 nm. Following further conjugation with PEI-AuNPs, the diameter of AuNPs-DOX-NDs increased to 368.10 ± 1.42 nm, indicating that the surface modification further increased the hydrodynamic size of the droplets. Zeta potential measurements showed that NDs and DOX-NDs were negatively charged, with values of −34.77 ± 0.42 mV and −31.67 ± 1.02 mV, respectively, while PEI-AuNPs displayed a positive potential of +31.77 ± 1.02 mV (Figure 2D). After conjugation, the zeta potential of AuNPs-DOX-NDs shifted to +36.37 ± 0.25 mV, further supporting successful modification by PEI-AuNPs.

The UV–Vis spectrum of DOX-NDs showed a characteristic absorption peak of DOX at approximately 480 nm (Figure 2E), indicating successful drug loading. Optimization of the initial DOX concentration is shown (Figure 2F,G). When the feed concentration increased from 0.05 to 0.20 mg/mL, the particle size changed only slightly, whereas a further increase to 0.30 mg/mL led to an obvious size increase. Considering both size stability and drug loading, 0.20 mg/mL was selected as the optimal DOX feed concentration, giving an encapsulation efficiency of 47.09% and a drug loading content of 2.86%.

After PEI-AuNPs conjugation, the UV–Vis spectrum of AuNPs-DOX-NDs showed a different absorption profile from that of DOX-NDs, with enhanced absorption around 520 nm and in the longer-wavelength region, including the NIR region near 808 nm (Figure 2H). This difference is mainly attributed to the localized surface plasmon resonance (LSPR) absorption of AuNPs, which typically appears around 520 nm for gold nanospheres and may extend toward longer wavelengths depending on nanoparticle size and surface environment. Therefore, the apparent shift in the major absorption region from below 500 nm in DOX-NDs to above 500 nm in AuNPs-DOX-NDs reflects the successful introduction of AuNPs rather than an inconsistency between the two spectra. These results further supported the successful construction of AuNPs-DOX-NDs with both DOX loading and AuNP surface modification.

The amount of PEI-AuNPs added was also optimized (Figure 2I,J). With increasing PEI-AuNP concentration, the main size-distribution peak gradually shifted to larger sizes and the Au content increased accordingly. However, a bimodal size distribution appeared at 30 mg/L, suggesting reduced colloidal stability. Therefore, 20 mg/L was chosen as the optimal addition concentration, at which the Au content in the final nanodroplets reached 28.77 ± 0.131 mg/L. Stability testing further showed that the particle size of AuNPs-DOX-NDs remained nearly unchanged during storage at 4 °C for 7 days, and the PDI stayed below 0.3 throughout the test period (Figure 2K), indicating acceptable storage stability.

To further confirm the successful incorporation of AuNPs into the AuNPs-DOX-ND formulation, complementary elemental characterization was performed by SEM-EDS and ICP-OES (Figure S1A–D). Representative SEM images and corresponding EDS elemental mapping showed a clear and markedly enhanced Au signal in AuNPs-DOX-ND compared with DOX-NDs (Figure S1A). A low Au background was also detected in the DOX-ND control; however, this was mainly attributable to the gold sputter coating applied during SEM sample preparation for EDS analysis, rather than intrinsic Au in the formulation itself. Consistent with the mapping results, the EDS sum spectrum of AuNPs-DOX-NDs displayed a distinct Au characteristic peak, whereas the Au signal in DOX-NDs was much weaker (Figure S1C). In addition, map-sum quantification showed a markedly higher Au content in AuNPs-DOX-NDs than in DOX-NDs (Figure S1D). Quantitative ICP-OES analysis further confirmed the presence of Au in the final formulation, with a Au concentration of 28.777 ± 0.131 mg/L (*n* = 3), while the detected P concentration was 0.213 ± 0.035 mg/L (*n* = 3) (Figure S1B). The phosphorus signal is consistent with the phospholipid-based droplet matrix. Taken together, these results provide direct qualitative and quantitative evidence for the successful incorporation of PEI-AuNPs into the AuNPs-DOX-ND formulation.

Taken together, these results show that AuNPs-DOX-NDs were successfully prepared through sequential loading of DOX and surface conjugation of AuNPs based on the PFH nanodroplet platform. The resulting formulation achieved co-loading of DOX and AuNPs while maintaining good dispersibility and stability.

### 3.2. Photothermal Performance of AuNPs-DOX-NDs

**Figure 3:**
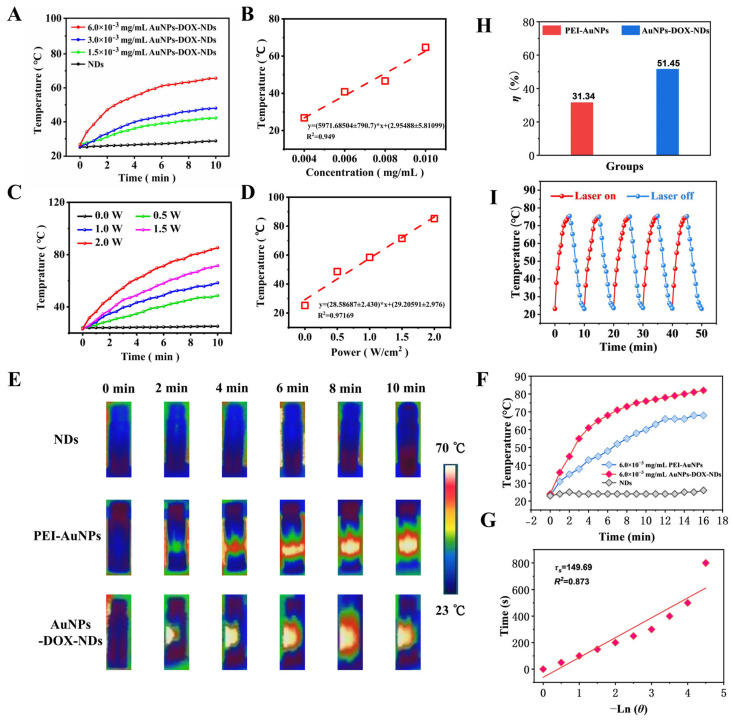
Photothermal performance of AuNPs-DOX-NDs. (**A**) Temperature–time heating curves of AuNPs-DOX-NDs, PEI-AuNPs, and blank NDs at different concentrations under 808 nm laser irradiation. (**B**) Linear fitting relationship between final temperature and gold concentration. (**C**) Temperature–time heating curves at different laser powers. (**D**) Linear fitting relationship between final temperature and laser power. (**E**) Infrared thermal images of AuNPs-DOX-NDs, PEI-AuNPs, and blank NDs at different time points (0–12 min) under 2.0 W/cm^2^ laser irradiation. (**F**) Comparative heating curves under the same laser conditions. (**G**) Photothermal conversion efficiency (*η*) comparison between PEI-AuNPs and AuNPs-DOX-NDs. (**H**) Linear fitting curve of −ln(*θ*) versus time during photothermal conversion efficiency calculation. (**I**) Photothermal stability testing curve under cyclic laser on/off conditions.

AuNPs-DOX-NDs showed a clear photothermal response under 808 nm laser irradiation. As the Au concentration increased, both the heating rate and the final equilibrium temperature increased accordingly, and a good linear relationship was observed (Figure 3A,B). Similarly, when the laser power density was increased from 0.5 to 2.0 W/cm^2^, the temperature rise became more pronounced, with ΔT showing a linear dependence on power density (Figure 3C,D). These results indicate that the photothermal effect of the system can be regulated by both Au content and laser power, which provides flexibility for subsequent experimental design.

To further compare the heating behavior, AuNPs-DOX-NDs (stock Au concentration: 28.777 mg/L) were diluted to an equivalent Au concentration of 6 μg/mL, and free PEI-AuNPs at the same Au concentration were used as a control. Infrared thermal imaging showed that under 808 nm laser irradiation (2.0 W/cm^2^) for 0–10 min, blank NDs displayed almost no temperature increase, free PEI-AuNPs gradually changed from blue-green to yellow, while AuNPs-DOX-NDs rapidly developed a red high-temperature region that expanded over time (Figure 3E). The quantitative temperature curves were consistent with these images and showed that AuNPs-DOX-NDs had a faster heating rate and reached a higher equilibrium temperature than free PEI-AuNPs at the same Au concentration (Figure 3F).

The photothermal conversion efficiency was calculated based on the cooling curve according to the method reported by Roper [21]. Briefly, the dimensionless driving-force temperature was defined as

*θ* = (*T* − *T_surr_*)/(*T_max_* − *T_surr_*), (3)

and −ln*θ* was plotted against the cooling time *t* (Figure 3G). The thermal time constant (*τs*) was determined to be 149.69 s from the reciprocal of the fitted slope, and the value of *hS* was obtained using

*hS* = *m_D_C_D_*/*τ_s_*. (4)

The photothermal conversion efficiency (*η*) was calculated as

*η* = [*hS*(*T_max_* − *T_surr_*) − *Q_dis_*]/[*I*(1 − 10^−*A*808^)]. (5)

Based on these parameters, the photothermal conversion efficiency of AuNPs-DOX-NDs was calculated to be 51.45%, which was markedly higher than that of free PEI-AuNPs (31.34%) (Figure 3H). In addition, repeated laser on/off cycling showed nearly overlapping heating and cooling curves over three cycles without obvious attenuation (Figure 3I), indicating good photothermal stability.

### 3.3. Phase Transition and Drug Release Under Temperature/Laser Regulation

**Figure 4:**
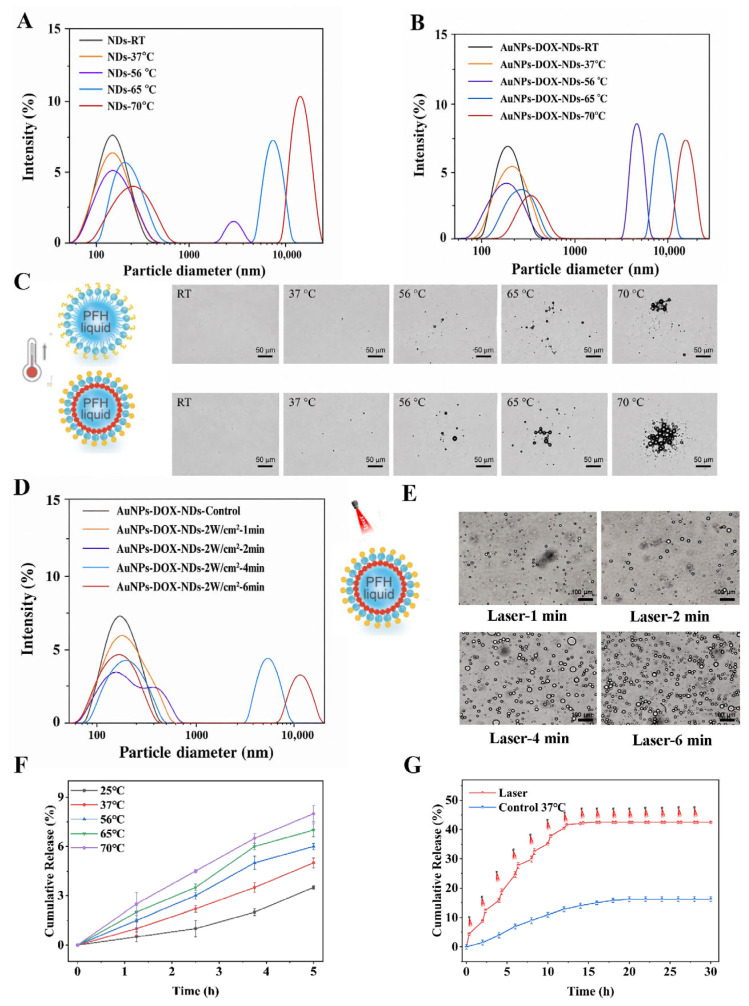
Phase transition and drug release behavior of AuNPs-DOX-NDs. (**A**) Particle size distribution curves of NDs at different temperature. (**B**) Particle size distribution curves of AuNPs-DOX-NDs at different temperatures. (**C**) Microscope images of NDs (upper row) and AuNPs-DOX-NDs (lower row) at different temperatures for observing temperature-induced liquid–gas phase transition. (**D**) Particle size distribution curves of AuNPs-DOX-NDs after 2.0 W laser irradiation for 1, 2, 4, and 6 min. (**E**) Microscope images of AuNPs-DOX-NDs under unirradiated and 2.0 W laser irradiation (1, 4, 6 min) conditions. (**F**) Cumulative drug release curves of AuNPs-DOX-NDs at different temperatures within 5 h. (**G**) Cumulative drug release curves of AuNPs-DOX-NDs under laser irradiation and 37 °C control conditions.

The phase-transition behavior of NDs and AuNPs-DOX-NDs under heat stimulation was first examined. As the temperature increased, the size distribution of both formulations gradually changed from a unimodal to a bimodal pattern (Figure 4A,B), suggesting progressive vaporization of the PFH core. Meanwhile, the overall rightward shift in the distribution peaks indicated the formation of larger particles or bubbles during the liquid–gas transition (Figure 4C). Correspondingly, the intensity of the nanoscale peak decreased, whereas the microscale peak became more evident.

The laser-triggered phase-transition behavior of AuNPs-DOX-NDs was further investigated. After 808 nm laser irradiation (2.0 W/cm^2^) for 1, 2, 4, and 6 min, the particle size distribution gradually changed from unimodal to bimodal (Figure 4D). In parallel, micrometer-sized bubbles could be directly observed under an optical microscope (Figure 4E). These findings suggest that laser irradiation induced PFH vaporization, leading to obvious volume expansion and disruption of the droplet structure, which may facilitate drug release.

To evaluate the release behavior of DOX, in vitro drug release experiments were carried out under different temperature and laser conditions. Under simple temperature elevation, DOX release increased only slightly. When the temperature rose from 25 °C to 70 °C, the cumulative release over 5 h increased from about 2% to about 10% (Figure 4F). In contrast, under 808 nm laser irradiation at 2.0 W/cm^2^, the release behavior changed markedly (Figure 4G). At this power density, the temperature of the AuNPs-DOX-NDs suspension rapidly exceeded 56 °C, which is sufficient to trigger the phase transition of PFH. In the non-irradiated group (37 °C), DOX release remained slow, and the cumulative release reached only about 15% at 24 h, indicating good drug retention under physiological conditions. By comparison, the laser-treated group showed a rapid release phase during the first 10–12 h, followed by a gradual plateau, with a final cumulative release of about 42%. More importantly, the release rate increased rapidly during laser-on periods and slowed down after the laser was turned off, showing a typical on–off pulsatile release behavior.

These results suggest that the release of DOX is mainly driven by photothermal-triggered phase transition rather than simple bulk heating. Under laser irradiation, AuNPs convert light energy into local heat, which induces PFH vaporization and structural disruption of the lipid shell, thereby accelerating DOX release. Once the laser is removed, the temperature decreases and the release rate slows accordingly. This externally controlled release pattern is more effective and controllable than passive thermal stimulation and may be advantageous for on-demand drug delivery.

### 3.4. Enhanced Combined Photothermal–Chemotherapeutic Effects of AuNPs-DOX-NDs in 4T1 Cells

The antitumor activity of AuNPs-DOX-NDs was evaluated in 4T1 cells. First, the biocompatibility of the drug-free carrier (AuNPs-NDs) was assessed. CCK-8 assay showed that AuNPs-NDs had good cytocompatibility in the concentration range of 0–100 μg/mL, although cell viability began to decline when the concentration reached 80 μg/mL or above (Figure S2A). Therefore, 70 μg/mL was selected as the maximum working concentration in the following experiments. Laser safety was also examined. In the absence of any material, cells exposed to 808 nm laser irradiation at 1.0 W/cm^2^ for 0, 2, 4, 6, 8, and 10 min maintained viabilities close to 100% (Figure S2B), indicating that the laser itself did not cause obvious cytotoxicity under the selected conditions.

To determine a suitable irradiation condition for the cell culture experiments, both laser safety and photothermal performance were evaluated. First, 4T1 cells were exposed to an 808 nm laser at 1.0 W/cm^2^ for different durations in the absence of any material. Cell viability remained high after 0–10 min of irradiation, indicating that laser irradiation alone did not cause obvious cytotoxicity under the tested conditions (Figure S2B). Next, temperature changes in wells containing free DOX, DOX-NDs, AuNPs-NDs, AuNPs-DOX-NDs, or medium alone were monitored by infrared thermal imaging. Only the AuNPs-ND and AuNPs-DOX-ND groups showed obvious heating during irradiation, whereas the DOX, DOX-ND, and control groups exhibited minimal temperature changes (Figure S2C,D). In the AuNPs-DOX-ND group, the temperature increased from 22 °C to 45.8 °C after 4 min of irradiation, indicating effective AuNP-mediated photothermal activation under the cell culture conditions. When the irradiation time was extended to 6 min, the temperature further increased to 53 °C, which might introduce excessive nonspecific thermal damage. Under the same conditions, the AuNPs-ND group reached 41.6 °C after 4 min. Therefore, 1.0 W/cm^2^ for 4 min was selected for the subsequent in vitro antitumor experiments as a moderate condition that balanced effective photothermal stimulation and experimental safety.

**Figure 5:**
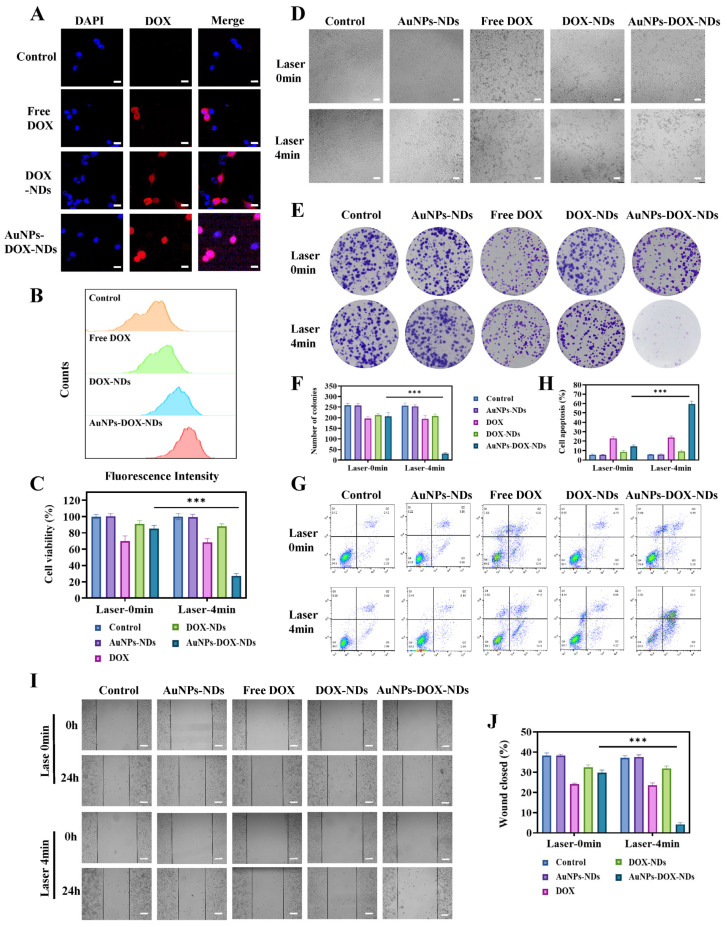
In vitro antitumor effects of AuNPs-DOX-NDs. (**A**) Confocal microscopy images of 4T1 cells after co-incubation with different treatment groups (control, AuNPs-NDs, free DOX, DOX-NDs, and AuNPs-DOX-NDs) with/without laser irradiation (scale bar = 25 μm). (**B**) Flow cytometry quantitative analysis of intracellular DOX fluorescence intensity. (**C**) CCK-8 assay of cell viability under different treatments with/without laser irradiation.(**D**) Bright-field morphological images of 4T1 cells under different treatments with/without laser irradiation (scale bar = 100 μm). (**E**,**F**) Colony formation assay of long-term proliferation inhibition. (**G**,**H**) Flow cytometry detection of apoptosis induction. (**I**,**J**) Wound healing assay of cell migration inhibition (scale bar = 100 μm). One-way ANOVA was performed and followed by Tukey’s post hoc multiple-comparison test. Statistical significance was indicated as *** *p* < 0.001.

The antitumor effects of different formulations were then compared. Without laser irradiation, free DOX showed moderate cytotoxicity, whereas DOX-NDs and AuNPs-DOX-NDs induced only limited cell damage, implying that DOX release from the nanocarriers was relatively slow in the absence of external stimulation. After laser irradiation, marked morphological changes, including cell rounding, detachment, and fragmentation, were observed only in the AuNPs-DOX-ND group, accompanied by a significant decrease in cell viability (Figure 5C,D).

This trend was further supported by colony formation and apoptosis analyses. Colony formation was most strongly inhibited in the laser-treated AuNPs-DOX-ND group (Figure 5E,F), indicating a strong long-term suppressive effect on cell proliferation. Flow cytometry also showed that this group had the highest apoptosis rate among all treatments (Figure 5G,H). In addition, wound healing assays demonstrated that laser-activated AuNPs-DOX-NDs significantly reduced wound closure compared with the other groups (Figure 5I,J), suggesting the effective inhibition of cell migration.

Overall, these findings indicate that laser-activated AuNPs-DOX-NDs produced an enhanced combined photothermal–chemotherapeutic effect in 4T1 cells, resulting in stronger inhibition of cell proliferation, survival, and migration than the corresponding non-laser or single-function treatment groups.

## 4. Discussion

TNBC is an aggressive breast cancer subtype that lacks widely effective molecular targets, and chemotherapy remains one of the main treatment options in clinical practice [1,3,22]. However, conventional chemotherapy is often limited by poor tumor selectivity, systemic toxicity, and insufficient drug accumulation or release at the tumor site. In this context, nanoplatforms that enable localized and controllable drug release while integrating multiple therapeutic functions are of considerable interest for improving TNBC treatment [3,4,23].

In this study, we developed a NIR-responsive phase-change nanodroplet system, AuNPs-DOX-NDs, for combined photothermal therapy and chemotherapy. Gold nanostructures for photothermal applications and perfluorocarbon phase-change droplets for stimulus-responsive delivery have both been widely investigated. However, compared with the extensive research framework established for ultrasound-responsive phase-change droplets, studies directly integrating gold nanostructures, perfluorocarbon phase-change droplets, and chemotherapeutic drug delivery remain relatively limited. In many previously reported systems, gold nanostructures have mainly been used as co-loaded photothermal/photoacoustic components or as shell/coating materials to facilitate laser-induced vaporization. In the present system, PEI-AuNPs were introduced onto the surface of PFH–DOX lipid nanodroplets as an interface-localized photothermal functional layer. This interfacial design allows AuNPs to convert 808 nm NIR light into localized heat at the droplet boundary and thereby influence the phase-transition behavior of the PFH core, rather than functioning only as passive photothermal additives.

A notable advantage of phase-change droplets is their inherent tunability. Unlike conventional nanocarriers, whose structures are generally fixed after formulation and whose drug release mainly relies on diffusion, degradation, or endogenous stimuli, PFH-containing droplets possess a metastable internal core that can undergo structural transformation under external energy stimulation. Surface functionalization with PEI-AuNPs further extends this tunable behavior from the more commonly studied ultrasound-triggered mode to an additional NIR-responsive mode. Accordingly, the carrier behavior in this system can be modulated by external laser parameters, including irradiation time, power density, and irradiation cycles, allowing more controllable phase-transition-associated drug release. From this perspective, the main significance of the present design lies not simply in combining photothermal therapy and chemotherapy within one platform, but in providing a phase-change carrier with enhanced external responsiveness and more actively regulatable drug-release behavior.

Nevertheless, the relatively large hydrodynamic diameter (~368 nm) may limit the systemic delivery efficiency of intact nanodroplets in vivo, particularly with respect to prolonged circulation, vascular extravasation, and deep intratumoral penetration after intravenous administration. In general, particles of this size are less favorable for passive diffusion through dense tumor interstitium, and their distribution may be more restricted to perivascular or relatively permeable tumor regions. Therefore, the current results mainly support the in vitro feasibility of this NIR-responsive phase-change system rather than its suitability for systemic delivery in the present form.

From a translational perspective, this platform may be more suitable for localized delivery strategies, such as local implantation, intratumoral administration, or integration into injectable hydrogels or postoperative cavity-filling materials, where prolonged blood circulation and deep penetration of intact droplets are less critical. Under such conditions, the relatively large particle size may even contribute to local retention at the administration site and help form a more stable drug depot for repeated external stimulation and on-demand release.

In addition, the therapeutic performance of this system may not depend entirely on the deep penetration of intact nanodroplets in their original size state. Upon NIR irradiation, AuNP-mediated local heating can induce PFH-related structural transformation and, under sufficiently strong conditions, phase transition with shell disruption, thereby triggering local DOX release. Once released, DOX is no longer constrained by the original carrier size and may diffuse more readily into adjacent tumor tissue. Moreover, the local physical effects associated with droplet phase transition, including volume expansion and structural disruption, may further influence the surrounding microenvironment and facilitate drug spread to nearby regions. However, these possibilities have not yet been directly evaluated in vivo, and further studies are still needed to determine the biodistribution, local retention, intratumoral transport, and therapeutic performance of this formulation in animal models.

The photothermal results showed that AuNPs-DOX-NDs had a photothermal conversion efficiency of 51.45%, which was clearly higher than that of free PEI-AuNPs (31.34%). One possible explanation is that the successful association of AuNPs with the droplet interface, as supported by elemental characterization, may favor local heat accumulation and reduce heat dissipation compared with freely dispersed nanoparticles [24]. In addition, the heating effect could be adjusted by changing either the Au concentration or the laser power, which is useful for controlling treatment intensity under different experimental conditions.

The irradiation condition used for the in vitro antitumor experiments (808 nm, 1.0 W/cm^2^, 4 min) was selected primarily on the basis of our own photothermal heating behavior and laser safety evaluation under the actual cell culture conditions, rather than being directly adopted from previous literature. Specifically, laser irradiation alone at 1.0 W/cm^2^ did not cause obvious cytotoxicity to 4T1 cells within the tested time range, indicating that this power density itself did not produce significant nonspecific damage. Under the same cell culture conditions, AuNPs-DOX-NDs showed a clear temperature increase during irradiation, reaching a moderate hyperthermic range after 4 min, whereas longer irradiation produced further heating that could increase the risk of excessive nonspecific thermal injury. Therefore, 4 min was considered a balanced exposure duration that was sufficient to produce effective AuNP-mediated photothermal stimulation while maintaining biological tolerability in the in vitro therapeutic experiments.

It should also be noted that the 15 min irradiation used in the photothermal characterization and release-related studies served a different purpose from the 4 min irradiation used in the cell experiments. The longer irradiation condition was intended to more fully reveal the heating behavior, structural response, and laser-triggered release capability of the formulation under relatively strong stimulation, whereas the shorter irradiation used in the biological experiments was chosen to provide a milder and safer treatment condition for evaluating antitumor efficacy. Therefore, these two irradiation settings should not be directly compared in terms of the extent of phase transition or drug release.

Importantly, the 4 min irradiation condition should not be interpreted as necessarily inducing complete PFH vaporization. Although more obvious phase-transition-related changes were observed under stronger heating conditions, the temperature-response, laser-response, and release data collectively suggest that some degree of early droplet structural response and laser-enhanced DOX release may already occur under the milder condition used in the cell experiments. This is reasonable because the activation behavior of phase-change droplets is influenced not only by the bulk boiling point of PFH, but also by droplet size, shell composition, interfacial effects, and local energy deposition [25,26]. In the present system, interface-localized AuNPs may generate localized heating near the droplet boundary under NIR irradiation, so the thermal response at the interface may be more pronounced than indicated by the macroscopic average temperature alone. Thus, 1.0 W/cm^2^ for 4 min was considered an appropriate compromise between therapeutic activation and experimental safety, although complete phase transition under this condition was not directly confirmed.

The release experiments further clarified the behavior of this system. Heating alone produced only a limited increase in DOX release, whereas laser irradiation induced much stronger and more controllable release, together with a typical pulsatile on–off pattern. Combined with the phase-transition data, these results suggest that the release mechanism is not governed simply by bulk thermal diffusion, but is more closely associated with AuNP-mediated local heating, which can promote PFH-related structural response and, under sufficiently strong irradiation, PFH vaporization and disruption of the lipid shell [24,27]. This process allows the laser to function as an external switch for drug release, which may improve local drug utilization while reducing unnecessary exposure to normal tissues [28].

The in vitro experiments also supported the design concept of this platform. The blank carrier showed acceptable cytocompatibility at the working concentration, and the selected laser condition itself did not cause measurable toxicity, which is consistent with previous reports on mild photothermal treatment windows [29]. In addition, cellular uptake of AuNPs-DOX-NDs was stronger than that of free DOX, and the PEI-modified surface may have contributed to this effect by enhancing interaction with the negatively charged cell membrane [30]. Increased intracellular accumulation of both DOX and AuNPs is beneficial for subsequent laser-triggered drug release and photothermal action.

Comparison among the treatment groups also helps explain the source of the enhanced combined therapeutic effect. AuNPs-NDs alone were able to produce a certain temperature increase, but their direct cytotoxicity remained limited, likely because mild hyperthermia mainly induces apoptosis rather than immediate necrosis. DOX-NDs contained the chemotherapeutic drug but lacked the photothermal component, and therefore did not show a strong advantage under laser exposure. By contrast, AuNPs-DOX-NDs combined both functions within one platform, enabling laser-triggered heating and enhanced DOX release at the same time. This dual action likely accounts for the strongest inhibition of viability, colony formation, and migration observed in 4T1 cells.

Nevertheless, this study still has several limitations. First, the current evaluation was restricted to in vitro experiments, and in vivo studies are still needed to assess biodistribution, tumor accumulation, therapeutic efficacy, long-term safety, and clearance behavior. Second, the in vitro biological evaluation was performed only in 4T1 tumor cells, and a non-cancerous cell-line control was not included. Therefore, the therapeutic selectivity of AuNPs-DOX-NDs toward tumor cells over normal cells has not yet been fully demonstrated. Future studies should include relevant non-cancerous cells, such as mammary epithelial cells or fibroblasts, to further evaluate formulation biosafety, tumor selectivity, and potential off-target cytotoxicity. These issues will be important for further assessing the translational potential of this NIR-responsive phase-change nanodroplet system.

In summary, we constructed a NIR-responsive AuNP-functionalized phase-change nanodroplet platform and demonstrated that interface-localized AuNPs enabled externally regulated PFH-related structural response, laser-triggered pulsatile DOX release, and enhanced combined photothermal–chemotherapeutic activity in 4T1 cells. These findings support the potential of this design as an externally controllable drug-delivery system for future local or image-guided therapeutic applications.

## 5. Conclusions

In this study, we developed a NIR-responsive gold-decorated phase-change nanodroplet system (AuNPs-DOX-NDs) composed of a PFH core, a DOX-loaded lipid shell, and surface-conjugated PEI-AuNPs for combined photothermal therapy and chemotherapy against TNBC. The nanodroplets showed a uniform morphology, successful Au incorporation as confirmed by SEM-EDS and ICP-OES, an average size of 368.10 ± 1.42 nm, good colloidal stability, and a photothermal conversion efficiency of 51.45% under 808 nm irradiation. Laser exposure triggered PFH vaporization and promoted controllable pulsatile release of DOX, which was much more pronounced than that induced by temperature alone. In vitro experiments in 4T1 cells further demonstrated enhanced intracellular drug accumulation, increased apoptosis, and stronger inhibition of proliferation and migration after laser activation. Overall, AuNPs-DOX-NDs showed promising enhanced combined photothermal–chemotherapeutic activity in 4T1 cells after laser activation and may serve as a potential stimulus-responsive platform for further evaluation.

## Data Availability

The data presented in this study are available from the corresponding author upon reasonable request.

## Supplementary Materials

The following supporting information can be downloaded at https://www.mdpi.com/article/10.3390/pharmaceutics18070816/s1, Figure S1. Characterization of AuNP-decorated DOX-loaded phase-change nanodroplets by ICP-OES, SEM, and EDS. (A) ICP-OES analysis of Au and P concentrations in AuNPs-DOX-NDs. (B) Representative SEM images, EDS overlay images, and Au elemental maps of AuNPs-DOX-NDs and DOX-NDs. (C) EDS sum spectra of AuNPs-DOX-NDs and DOX-NDs. (D) Map-sum EDS quantification of elemental composition in AuNPs-DOX-NDs and DOX-NDs. Figure S2. Supplementary characterization. (A) Effect of blank carrier AuNPs-NDs at different concentrations (0–100 μg/mL) on 4T1 cell viability. (B) Effect of 808 nm laser irradiation (1 W/cm^2^) for different durations (0–10 min) on 4T1 cell viability without materials. (C,D) Infrared thermal imaging or temperature rise results of different treatment groups under cellular experiment laser parameters. One-way ANOVA was performed and followed by Tukey’s post hoc multiple-comparison test. Statistical significance was indicated as * *p* < 0.05, ** *p* < 0.01, and *** *p* < 0.001.
