# Supplementary material for: NIR-Responsive Gold-Decorated Phase-Change Nanodroplets for Photothermal-Triggered Pulsatile Doxorubicin Release and Enhanced Combined Photothermal-Chemotherapy in Triple-Negative Breast Cancer
Source: Pharmaceutics. 2026 Jun 30;18(7):816. doi: 10.3390/pharmaceutics18070816 (PMC13415136; doi:10.3390/pharmaceutics18070816)
Supplement: Supplementary file 1 [file pharmaceutics-18-00816-s001.zip › pharmaceutics-4385194-supplementary.pdf]

NIR-Responsive Gold-Decorated Phase-Change Nanodroplets for Photothermal-Triggered Pulsatile Doxorubicin Release and Synergistic Therapy against Triple-Negative Breast Cancer

Luyao Ma 1, Fulai Chen 1, Qinghao Xu 1, Jianwei Yu 1, Yang Liu 1,* and Lei Duan 1,*

1 School of Biomedical Engineering and Informatics, Nanjing Medical University, Nanjing 211166, China
* Correspondence: [lyseubme@gmail.com] (Y.L.); LeiDuan_NJMU@163.com (L.D.)

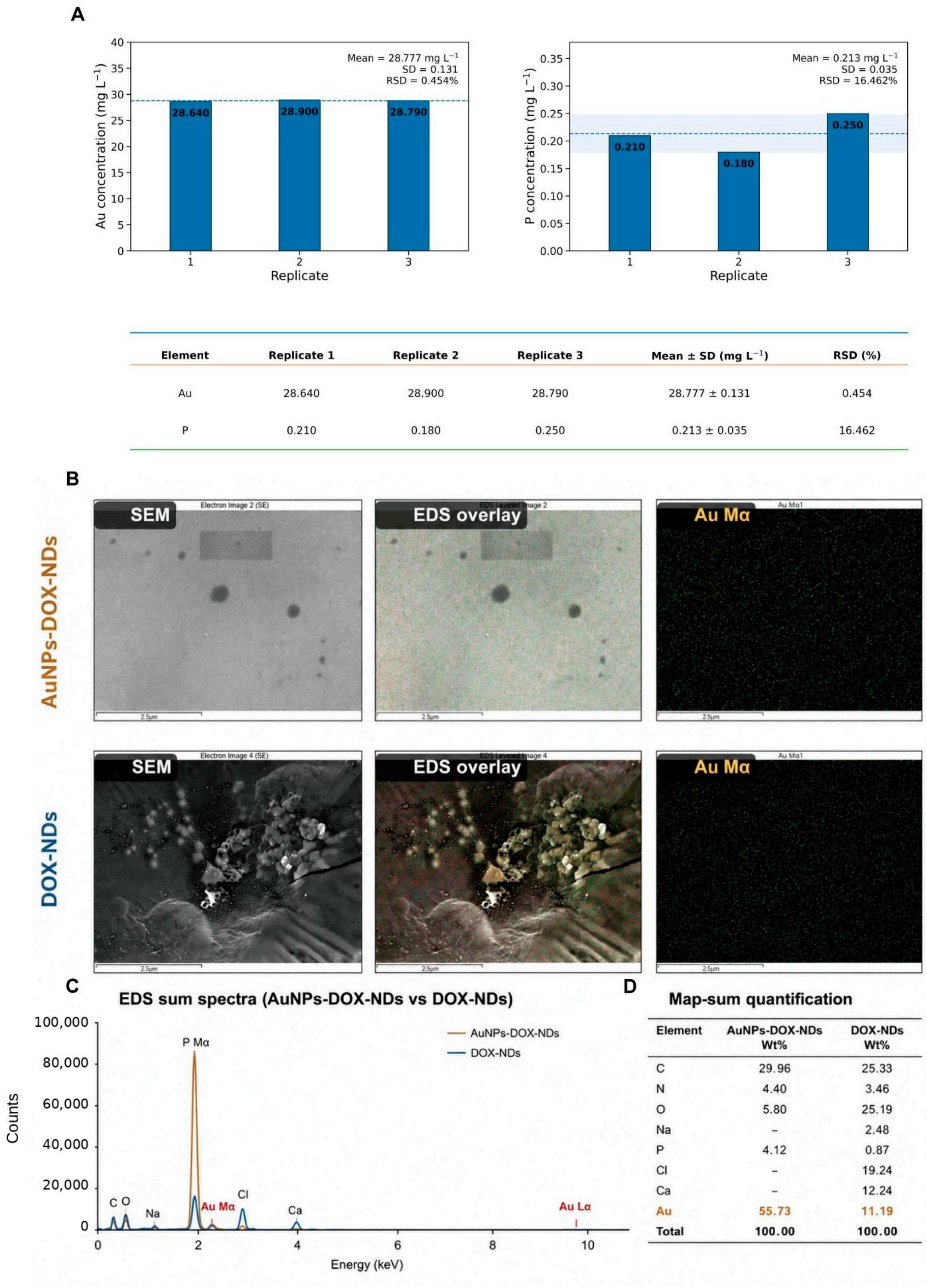

Figure S1. Characterization of AuNP-decorated DOX-loaded phase-change nanodroplets by ICP-OES, SEM, and EDS. (A) ICP-OES analysis of Au and P concentrations in AuNPs-DOX-NDs. (B) Representative SEM images, EDS overlay images, and Au elemental maps of AuNPs-DOX-NDs and DOX-NDs. (C) EDS sum spectra of AuNPs-DOX-NDs and DOX-NDs. (D) Map-sum EDS quantification of elemental composition in AuNPs-DOX-NDs and DOX-NDs.

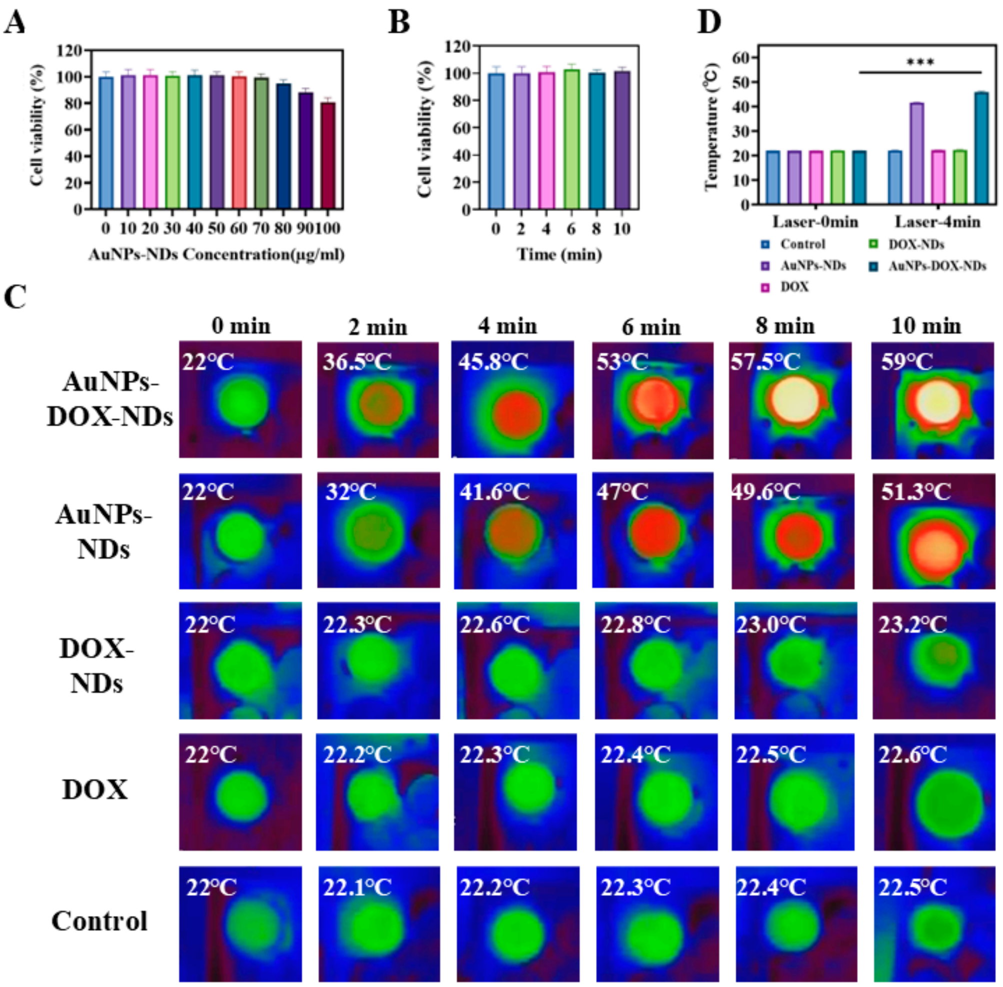

Figure S2. Supplementary characterization. (A) Effect of blank carrier AuNPs-NDs at different concentrations (0-100 μg/mL) on 4T1 cell viability. (B) Effect of 808 nm laser irradiation (1 W/cm²) for different durations (0-10 min) on 4T1 cell viability without materials. (C, D) Infrared thermal imaging or temperature rise results of different treatment groups under cellular experiment laser parameters. One-way ANOVA was performed and followed by Tukey's post hoc multiple-comparison test. Statistical significance was indicated as * $p < 0.05$, ** $p < 0.01$, and *** $p < 0.001$.
